# The Current and Potential Therapeutic Use of Metformin—The Good Old Drug

**DOI:** 10.3390/ph14020122

**Published:** 2021-02-05

**Authors:** Józef Drzewoski, Markolf Hanefeld

**Affiliations:** 1 Central Teaching Hospital of Medical University of Lodz, 92-213 Lodz, Poland; 2 Medical Clinic III, Department of Medicine Technical University Dresden, 01307 Dresden, Germany; hanefeld.radebeul@t-online.de

**Keywords:** metformin, pleiotropic effect, oxidative stress, inflammation, cardioprotection, nephroprotection, polycystic ovary syndrome, non-alcoholic fatty liver disease, gestational diabetes, type 1 diabetes, cancer, longevity

## Abstract

Metformin, one of the oldest oral antidiabetic agents and still recommended by almost all current guidelines as the first-line treatment for type 2 diabetes mellitus (T2DM), has become the medication with steadily increasing potential therapeutic indications. A broad spectrum of experimental and clinical studies showed that metformin has a pleiotropic activity and favorable effect in different pathological conditions, including prediabetes, type 1 diabetes mellitus (T1DM) and gestational diabetes mellitus (GDM). Moreover, there are numerous studies, meta-analyses and population studies indicating that metformin is safe and well tolerated and may be associated with cardioprotective and nephroprotective effect. Recently, it has also been reported in some studies, but not all, that metformin, besides improvement of glucose homeostasis, may possibly reduce the risk of cancer development, inhibit the incidence of neurodegenerative disease and prolong the lifespan. This paper presents some arguments supporting the initiation of metformin in patients with newly diagnosed T2DM, especially those without cardiovascular risk factors or without established cardiovascular disease or advanced kidney insufficiency at the time of new guidelines favoring new drugs with pleotropic effects complimentary to glucose control. Moreover, it focuses on the potential beneficial effects of metformin in patients with T2DM and coexisting chronic diseases.

## 1. Introduction

Metformin has served people with T2DM for more than six decades. Its important role in the management of chronic hyperglycemia and its consequences were best demonstrated by the results of the United Kingdom Prospective Diabetes Study (UKPDS), a multicenter, randomized, prospective study with a median follow-up of 10.7 years. This landmark clinical trial showed that only metformin, independently of the glucose-lowering effect, significantly reduced diabetes-related death, myocardial infarction, any diabetes-related endpoints, and all-cause mortality in 342 overweight/obese people with newly diagnosed T2DM and low cardiovascular (CV) risk [1]. By contrast intensive glucose control with sulfonylureas and insulin had no significant effect on major adverse cardiovascular events (MACE) (1). 

Importantly, a continued beneficial effect after metformin therapy on vascular complications of diabetes was observed in a 10-year post-trial follow-up of the UKPDS [2]. Metformin has also been shown to be effective in secondary prevention of cardiovascular outcomes [3,4]. The cardioprotective properties of metformin were also observed in several retrospective, non-randomized clinical trials with mostly relatively short duration. However, owing to differences in design and methodology the results of these studies, are not easy to interpret and the real impact of metformin on the cardiovascular system remains uncertain.

The preference for metformin over other available oral hypoglycemic agents in the initiation of T2DM therapy has been based predominately on the results of the UKPDS. Moreover, the leading role of metformin as first line drug in the treatment of newly diagnosed T2DM is supported by more than 60 years of very good experience with this drug in everyday clinical practice. However, according to the principles of evidence-based medicine, the strongest arguments supporting any given drug as the drug of the first choice in the management of any disease, should be provided by the results of meta-analyses and systematic reviews of randomized, high quality, controlled clinical trials. In the case of metformin, the findings from the majority of such studies have demonstrated substantial clinical benefits, including significant antihyperglycemic effect, relatively good tolerance and very low risk of hypoglycemia. Moreover, what is of particular importance, a desirable impact of this drug on the cardiovascular system and modifiable cardiovascular risk factors (obesity, insulin resistance (IR), hyperglycemia, hyperinsulinemia and dyslipidemia) has been demonstrated in numerous studies and meta-analyses [5,6,7,8]. However, significant cardioprotective effects of metformin on the cardiovascular system have been questioned by some researchers and clinicians [9,10,11]. Nevertheless, already in 2006 the American Diabetes Association (ADA) and the European Association for the Study of Diabetes (EASD) recommended—in a joint statement—the use of metformin along with diet and exercise as the initial pharmacologic intervention in subjects with T2DM [12]. The most recent ADA 2020 Standards of Care reiterated his position regarding the initiation of therapy in T2DM pointing out that metformin together with the lifestyle intervention should be continued as long as it is tolerated and not contraindicated [13]. Various other international and national guidelines also recommend prescription of this medication to control hyperglycemia in people with newly diagnosed T2DM.

Rapidly growing evidence of cardiorenal protection from large cardiovascular outcome trials (CVOTs) with some representatives of the newer classes of antidiabetic agents, including sodium-glucose co-transporter 2 inhibitors—(SGLT2 inh.)—empagliflozin, canagliflozin and dapagliflozin, glucagon-like peptide-1 receptor agonists (GLP-1Ras)—liraglutide, semaglutide has revealed significant glucose independent reduction in (MACE), including cardiovascular death, non-fatal myocardial infarction, non-fatal stroke and heart failure requiring hospitalization for unstable angina or heart failure (HF) [14,15,16,17]. Furthermore, it was reported that SGLT-2 inh. and GLP-1RAs medications may reduce the risk of kidney disease progression [18,19]. Spectacular results of these very well-designed studies with thousands of participants with T2DM and coexisting cardiorenal diseases when compared with not entirely convincing studies with metformin have opened a hot debate whether metformin should still occupy the leading position among antidiabetic drugs used to treat T2DM [9,10,11,20]. In a recent update to the previous recommendations for the management of hyperglycemia in type 2 diabetes, ADA and EASD advise that—after failure of metformin monotherapy—early implementation of GLP-1RAs or SGLT2 inh. could be considered to reduce the risk of MACE or chronic kidney disease (CKD) independently of baseline glycated hemoglobin A1c (HbA1c) or individualized HbA1c target [21].

This review primarily focuses on the pleiotropic activity of metformin and its favorable effects in different clinical conditions. It also presents some arguments that it is probably too early to replace metformin with newer hypoglycemic drugs as an initial pharmacologic therapy in newly diagnosed T2DM. 

## 2. A Brief History of Metformin

The long and turbulent history of metformin is closely linked to *Galega officinalis*, a traditional herbal medicine (also known as—among other names—as goat’s rue and French lilac) that was used since the Middle Ages to treat symptoms of what we now know to be diabetes [22,23]. The herb is a rich source of alkaloids, including guanidine (aminomethanamidine) and the less toxic galegine (isoamyleneguanidine). The glucose- lowering activity of guanidine was already demonstrated in experiments on rabbits by Watanabe in 1917 [24]. The pioneering synthesis of metformin was performed in 1922 by two Irish chemists, Werner and Bell, but it was not used for a long time [25]. The chemical structure of galegine moiety was described by Barger and White in 1923 [26]. Preclinical experiments with galegine—a less toxic guanidine-like alkaloid—revealed that in rabbits and dogs the hypoglycemic effect was too profound, leading to their death [27]. The results of the first human studies published in 1927 by Muller and Reinwein showed that the hypoglycemic effect of galegine was marked in individuals with diabetes and, interestingly, only mild in subject with normoglycemia [28]. Subsequent research confirmed these observations but the variability of glucose concentration, short duration of hypoglycemic effect and poor tolerance limited its clinical utility. To eliminate these drawbacks a series of guanidine analogues were synthesized. It was soon proved that molecules with two guanidines (biguanidines) in the same structure: metformin (1,1-dimethylbiguanide), phenformin (phenylethylbiguanide) and buformin (buthylbiguanide) have a greater hypoglycemic effect than those containing only one guanidine (monoguanidines). Until the 1940s, metformin was forgotten as an agent possessing weaker hypoglycemic activity than phenformin and buformin. Accidently, during clinical studies conducted at the time of the Second World War with a new antimalarial agent having a biguanide structure, a decrease in blood glucose concentrations was observed. This response to the tested compounds inspired Jean Stern, a French clinician, to assess the usefulness of metformin in the treatment of adult-onset diabetes. The results of his studies showing that oral administration of metformin effectively lowered glycemia in T2DM without causing hypoglycemia and lactic acidosis, were published already in 1957 and 1958 resp. [29,30]. Subsequent to the reports by Stern, metformin was introduced into the UK and other European countries in 1958 for the treatment of T2DM. Despite the positive information coming from Europe about good clinical effects of metformin, the agent was approved by the FDA for use in the United States only in 1994 and put on the marked one year later. The main cause of the delayed decision by the FDA was concern about increased risk of lactic acidosis and cardiovascular side effects associated with buformin and phenformin.

Since the introduction of metformin to T2DM therapy, countless number of patients have been treated successfully with this globally available medication having a favorable risk/benefit profile recommended by IDF guidelines as first line drug [31]. Hence, it is not surprising that metformin is still the most commonly prescribed oral antidiabetic medication worldwide with the prescription rate of 45–50% of all prescriptions and taken by over 150 million people each year [31,32,33]. Long-term positive experience with the use of metformin, strong evidence of clinical efficacy, safety, high adherence rate, low cost, general availability and cost-effectiveness were behind the decision of the World Health Organization to put it on the list of essential medicines: “medicines that satisfy the priority of health care needs of the population” [34].

## 3. Pleiotropic Effects of Metformin

### 3.1. Effects of Metformin on Glucose Metabolism

Despite intensive research the molecular mechanisms of metformin action have yet to be fully understood. Nevertheless, the knowledge that has accumulated so far indicates that they are unique and that their clinical and metabolic effects are extremely beneficial for the patient with T2DM. Briefly, in view of the evidence available, metformin interacts with multiple targets and signaling pathways within different cells thereby influencing many physiological and pathological processes, particularly associated with insulin resistance (IR) [5,35,36,37,38,39]. The most classical effect of metformin is reduction of hyperglycemia and alleviation of its clinical symptoms. The drug’s action results primarily from the inhibition of hepatic gluconeogenesis leading to a reduction of hepatic glucose output. The second important effect of metformin is an improvement of insulin signaling leading to subsequent increase of skeletal myocyte glucose uptake [38,40,41,42,43,44,45,46,47,48]. In animal studies it was demonstrated that metformin reduces hepatic glucose production by 50 to 60% [47]. Such a strong influence of metformin on liver gluconeogenesis has been confirmed in human studies. Their results showed that treatment of patients with T2DM with metformin resulted in over 30% decrease in this process [43,44,48].

It has been established that AMP-activated protein kinase, AMPK, is a critical energy sensor of the cell and cellular regulator of glucose, lipids and protein metabolism [49,50]. Multiple studies have shown that inhibition of hepatic glucose production by metformin is most likely possible both through AMPK-dependent mechanism and AMPK—independent mechanism [35,38,39,51,52,53]. Zhou et al. were the first to demonstrate in a series of experiments on isolated rat hepatocytes and rat liver that metformin affects the formation of functional AMPK heterotrimeric complex which is subsequently phosphorylated by upstream liver kinase B1 (LKB1). Phosphorylation of this enzyme is required for metformin’s inhibitory effect on glucose production by hepatocytes and for metformin stimulated glucose uptake by skeletal muscle [54]. The effect of metformin on AMPK activation was confirmed by Shaw et al. who noted that hepatic knockout of LKB1, an enzyme necessary for AMPK phosphorylation, abolishes the anti-hyperglycemic effect of metformin in mice fed a high-fat diet [55].

Studies on isolated mitochondria, submitochondrial particles, mitochondrial membranes and isolated mitochondrial respiratory chain have shown that reduction of gluconeogenesis by metformin in hepatocytes can also be attributed to a mild and transient inhibition of the mitochondrial respiratory chain of complex I [56,57,58,59,60]. Mitochondrial complex I inhibition by metformin, preventing mitochondrial adenosine triphoshate (ATP) synthesis, leads to an increase of adenosine monophosphate (AMP) level in the cell. Subsequently, AMP binds to one subunit of the AMPK making it more susceptible to phosphorylation by LKB1. Activated AMPK enhances insulin sensitivity of the liver and switches hepatocytes from an anabolic pathway, such as gluconeogenesis, fatty acid and protein synthesis, to a catabolic pathway, such as glycolysis and fatty acid oxidation consuming less energy and restoring energy balance [35,40,52,53,54,61]. As a result of improving insulin signaling and insulin sensitivity metformin increases glucose uptake and utilization in skeletal muscles thereby i regulating glycemic control in people with dysglycemia [62,63,64,65]. In turn, increasing the insulin sensitivity of adipose tissue by metformin inhibits lipolysis and reduces the release of free fatty acids [FFA] from adipocytes and their accumulation in the liver and other organs [35,65,66,67]. The inhibition of the mitochondrial respiratory chain of complex I by metformin in primary hepatocytes and animal models was observed at supra-pharmacological concentrations of metformin, 10–100 times higher than maximal therapeutic concentrations of the drug in the blood of T2DM patients [32,51,57]. Importantly, it has been reported that low metformin concentrations which are within the range of its therapeutic concentrations are also able to activate AMPK without inhibiting the mitochondrial respiratory chain of complex I and altering the AMP/ATP ratio [32,51,54,68]. Therefore, apart from AMPK dependent mechanisms of anti-hyperglycemic effects of metformin, other putative mechanisms of glucose homeostasis regulation by metformin have been proposed. These mechanisms include direct allosteric inhibition of the key mitochondrial gluconeogenesis enzymes in hepatocytes, ATP depletion, decreasing production of cyclic AMP by adenyl cyclase, increasing glucagon-like peptide-1 (GLP-1) secretion by enterocytes, decreasing activity of (dipeptidyl peptidase-4 (DPP-4), the enzyme responsible for inactivating GLP-1 in circulation and tissues, modulation of bile acid recirculation and metabolism, and improved action of intestine-pancreas axis by modifying the composition of the intestinal microbiota, modulation of hepatic redox potential by alteration in the glycerol-phosphate shuttle and blocking 1,6-biphoshatase-1 in the liver, inhibition of duodenal AMPK signaling and mechanism involving the lysosome, rather than the mitochondrion [39,40,53,69,70,71,72,73,74,75,76,77,78,79,80]. Recently, there has been particular interest in the effects of metformin on the gut. The results of several recent studies suggest that metformin increases glucose intestinal absorption and glucose utilization in enterocytes limiting the access of glucose to the bloodstream [71,72,73].

Insulin resistance is one of the major pathogenic mechanism of T2DM and metformin action is directed to the improvement of insulin sensitivity, especially in the liver and muscle [35,53,81,82,83,84]. The reduction of IR may be due to two different mechanisms. The direct effect of the drug on insulin sensitivity in hepatocytes occurs via the activation of AMPK, resulting in increased insulin receptor expression and tyrosine kinase activity with subsequent inhibition of gluconeogenesis and an increase in glycogen synthesis and beta oxidation of FFAs in the liver [35,38,40,83,84,85]. Activation of AMPK in the skeletal muscle by metformin promotes the recruitment and activity of glucose transporter 4 (GLUT4) transporters and enhances the uptake of glucose from the bloodstream and its utilization in anaerobic glycolysis [53,64,86,87]. The effect of metformin in adipose tissue through AMPK activation results in increased activity of glucose transporters (GLUT1 and GLUT4)], and increased fatty acids oxidation and lipolysis inhibition. Furthermore, metformin reduces the level of FFAs, proinflammatory cytokines and the outflow of several hormones from adipocytes. As a result, lipid accumulation in the liver is inhibited and insulin sensitivity of this organ improves [35,87,88,89,90].

The indirect effect of metformin on IR may arise as a consequence of reduced glucotoxicity and lipotoxicity [90,91,92]. A large number of preclinical and clinical studies have proven that the normalization of chronic hyperglycemia and hyperlipidemia through metformin-dependent improvement in insulin sensitivity can significantly reduce the destructive effects of these metabolic defects in many organs including the cardiovascular system, brain, liver, eyes and pancreas [1,2,3,4,93,94]. That is why early prevention of these metabolic disturbances and their harmful aftermath by metformin treatment can diminish the risk of beta cell damage and chronic diabetic complications [94,95,96]. At this point, it should be noted that the direct effect of metformin on IR, a unique feature of this drug, makes possible, at least at this moment, potentially broader therapeutic use of it (e.g., prediabetes, polycystic ovary syndrome (PCOS)) than of any of the newer hypoglycemic drugs [36,38,53,94].

### 3.2. Pleiotropic Effects of Metformin beyond Glucose Control

T2DM is a complex disease associated with an increasing risk of some pathological conditions, including obesity, metabolic syndrome, atherosclerotic cardiovascular diseases, certain types of cancer, non-alcoholic fatty liver disease (NAFLD), neurodegenerative diseases, PCOS, dementia and metabolic complications of non-retroviral infection [5,37,38,39,40,41,42]. Growing evidence suggests that metformin may substantially inhibit or delay their progression and improve prognosis, when added to the standard therapy of all of the above-mentioned diseases [36,41,94,97,98,99,100,101,102]. Moreover, metformin is sometimes used as an adjunct agent, together with insulin, to treat patients with type 1 diabetes [103,104].

**Figure 1:**
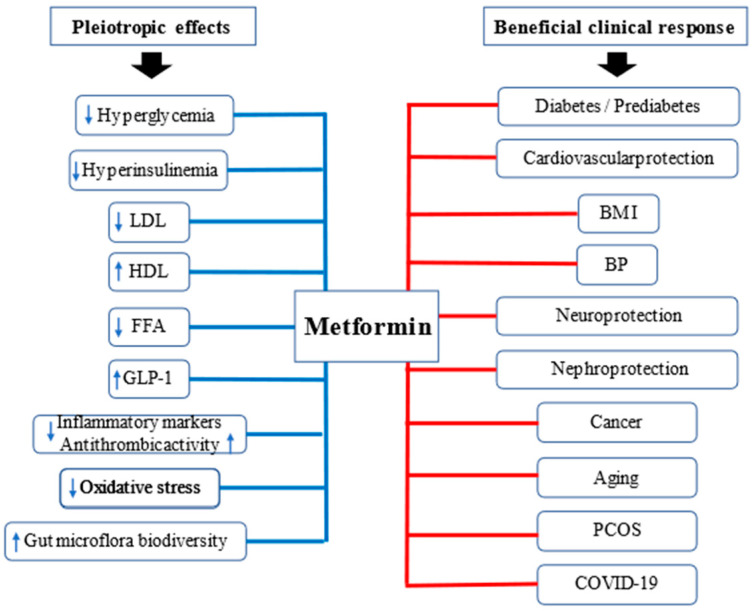
Clinical benefits of pleiotropic effects of metformin. Arrow-up—increase, arrow-down—decrease.

Metformin acts not only as a glucose-lowering drug but exhibits additional benefits, including moderate anti-inflammatory and anti-oxidative effects [93,105,106]. Hyperglycemia-related inflammation and oxidative stress are closely associated with IR and all these abnormalities affect beta cell leading to its secretory dysfunction and increased apoptosis resulting in T2DM development and progression [41,105,106]. Moreover, studies show that metformin slightly reduces body mass and arterial blood pressure in overweight and obese people [107,108,109]. It may also improve lipid profile and balance between profibrinolytic and anti-thrombotic factors [5,110]. However, recently the results of research showing potential anti-neoplastic and anti-ageing effects of metformin are of particular interest to scientists and clinicians [36,37,111,112]. The consequences of the pleiotropic effects of metformin are depicted in Figure 1.

#### 3.2.1. Metformin and Inflammation

The mechanisms responsible for diabetes–related inflammation and the role of metformin in inhibiting this process are extensively reviewed in refs [105,106].

Numerous studies using various animal and human cells (e.g., bovine aortic endothelial cells or human umbilical vein endothelial cells) have revealed that metformin suppresses hyperglycemia– related low–grade inflammation through the inhibition of nuclear factor kappa B (NF-κB) signaling cascade in various cells via AMPK- dependent and AMPK—independent pathways. Inhibition of NF-κB by AMPK reduces the expression of pro-inflammatory cytokines such as interleukin 1β, interleukin 6 and tumor necrosis factor alfa (TNFα). On the other hand, the drug increases the synthesis of anti-inflammatory cytokines (IL-4 and IL-10). Metformin is also capable of inhibiting chronic inflammation indirectly through improving insulin sensitivity, controlling hyperglycemia, advanced glycation end-products (AGEs)formation, body mass loss and diabetic atherogenic dyslipidemia [38,94,105,106,113]. The evaluation of the anti-inflammatory effects of metformin in people with prediabetes and T2DM have shown that treatment with this agent resulted in decreased plasma insulin level, plasminogen activator type 1 (PAI-1) antigen, C- reactive protein (CRP) and fibrinogen [114]. Krysiak and Okopień have shown a suppressive effect of metformin on the release of pro-inflammatory cytokines from monocyte and lymphocyte taken from patients with impaired glucose tolerance [115,116].

#### 3.2.2. Metformin and Oxidative Stress

Accumulating evidence from experimental and clinical studies indicates that chronic hyperglycemia is associated with elevated levels of free radicals, including extremely reactive oxygen species [ROS]. Excessive production of free radicals and the subsequent decline in cellular antioxidant defense leads to the oxidation of cell constituents, particularly lipids, proteins and DNA [117]. Consequently, oxidative stress promotes the injury and apoptosis of all body cells, including beta cells leading to the development of diabetes and its chronic complications. It was reported that metformin alleviates oxidative stress and endoplasmatic reticulum stress (ER) which are also responsible for pancreatic β-cells destruction [95]. It has been proven both in experimental and human studies that metformin improved the antioxidant status [93,95,96,118,119,120,121]. The mechanism of the antioxidant effect of metformin has not been fully clarified yet. However, as oxidative stress, inflammation, and IR are all involved in AMPK signaling, the potential benefits of metformin therapy in T2DM may be the results of this enzyme’s activation. Interestingly, Singh et al. have demonstrated in a prospective clinical study that insulin sensitizers, metformin and pioglitazone, have different anti-oxidant potential. Their findings indicate that metformin significantly decreases malondialdehyde (MDA) level and strengthens the anti-oxidant defense system (SOD). Pioglitazone significantly reduced MDA, but failed to raise the SOD level. This may suggest that metformin has a more extensive antioxidative effect than pioglitazone [122].

## 4. Clinical Aspects of Metformin

The aforementioned pleiotropic activities of metformin justify its leading position in the treatment of T2DM and opens new possibilities for using this exceptional medication prevention and management of various diseases (Table 1).

### 4.1. Type 2 Diabetes

After more than 60 years of clinical use the key role of metformin in the management of chronic hyperglycemia in T2DM may be said to have been proven beyond question. An uncountable number of clinical studies and real-world practice have strongly demonstrated its effectiveness, safety and good tolerance in monotherapy as well in combination with other glucose -lowering drugs. Therefore, metformin use along with diet and exercise as the initial pharmacologic intervention in subjects with T2DM is still recommended by ADA and EASD [12,13]. Since the role of metformin in T2DM has been extensively reviewed in the current literature, we decided to focus on other potential therapeutic applications of this unique drug. 

### 4.2. Prediabetes

The prevalence of prediabetes is rapidly increasing worldwide and according to the International Diabetes Federation 453.8 million of the world’s population will have impaired glucose tolerance [123]. People with prediabetes are at a high risk of progressing to T2DM and are prone to develop cardiovascular disease, including coronary heart disease and HF, cardiovascular death and stroke [124]. These individuals are often overweight or obese with elevated IR. Therefore, the use of metformin—an agent which significantly improves insulin sensitivity and modestly reduces body weight—to inhibit the progression or delay of early glucose metabolism disturbances—is understood when lifestyle intervention fails [125]. Furthermore, it is rational to consider metformin use in patients already exhibiting early stages of microvessel disease and fatty liver.

The efficacy of metformin to delay or prevent the conversion of prediabetes to diabetes has been proven in the landmark study—Diabetes Prevention Program (DPP)—which is the largest randomized clinical trial examining metformin use for diabetes prevention. The aim of this study was to assess whether intensive lifestyle intervention and metformin may prevent or delay the onset of T2DM in people at high risk of diabetes. The results of this trial showed that after 2.8 years of follow-up the incidence of diabetes was 58% lower in the lifestyle intervention and 31% lower in the metformin group in comparison with the placebo group [126]. The role of metformin in patients with stable coronary heart disease induced by dysglycemia and insulin resistance was assessed in the Codyce Multicenter Prospective study. The findings of this study indicate that, by decreasing hyperglycemia and insulin resistance, metformin may improve the endothelial function and reduce the high risk of MACE in people with glucose metabolism disturbances [114,125].

Summarizing the results from 40 studies on metformin use for diabetes prevention among people at higher risk published between 1998 and 2017 Moin et al. could conclude that the agent is effective, safe, tolerable, and cost effective. Therefore, they suggest increasing use of metformin in the real-world practice, particularly in obese persons with BMI ≥ 35 kg/m^2^, with elevated fasting glucose concentrations and HbA1c levels between 5.7–6.4% and women with a history of gestational diabetes [127]. Based upon the positive results of many studies, metformin is the only anti-diabetic agent recommended by the ADA to consider for the prevention of T2DM in people with prediabetes [128]. The use of the drug is permitted by low in some countries (e.g., Poland, Turkey, Philippines and the United Kingdom) [125]. To the best of our knowledge none of the newer anti-diabetic medications is recommended and used for the primary prevention of diabetes.

### 4.3. Type 1 Diabetes

Metformin is quite often used off-label as an add-on to insulin in T1DM because it has been observed that it may improve the whole-body and peripheral IR in young diabetics with obesity [129,130]. Therefore, until recently the rationale for it use was mainly based on the reduction in insulin-dose requirement observed in some clinical studies [130]. The first clinical studies evaluating the influence of metformin on glycemic control in patients with T1DM were performed in the mid-1980s with rather disappointing results [131]. However, studies carried out in the following years provided more optimistic results. Some of them have shown that addition of metformin to insulin resulted in a reduction in insulin requirement with or without improvement in HbA1c [132,133,134]. Vella et al. reported the results of a meta-analysis of the five randomized clinical trials assessing the role of metformin in T1DM. They found a significant reduction in insulin dose requirement but without lowering HbA1c [135]. A multicenter clinical trial performed in the USA in 2013/2014 aimed at assessing the effect of the addition of metformin in a dose of 2000 mg per day, to basal-bolus insulin in 140 overweight/obese adolescents with poorly controlled T1DM. At the end of the six-month-treatment with insulin plus metformin there was not improvement in glycemic control but insulin dose requirement and BMI were significantly reduced in comparison to placebo. However, the use of metformin was associated with significantly higher rate of gastrointestinal adverse effects. The authors of this trial concluded that these results do not support prescribing metformin to obese adolescents with T1DM to improve glycemic control [136].

Surprisingly, the data from the REducing with MetfOrmin Vascular Adverse Lesions (REMOVAL) trial have shown that metformin in adults aged 40 years or older with long-durationT1DM can reduce weight and also moderately reduce insulin requirement, as well as LDL-cholesterol levels, and, what is of particular importance, it inhibits the progression of atherosclerosis. These findings create a new perspective for the wider use of metformin in T1DM, especially in middle-aged overweight/obese people with diabetic dyslipidemia, and receiving high doses of insulin [104].

By contrast to metformin, none of the newer hypoglycemic agents is used in the treatment of T1DM. However, a few short-term randomized trials with SGLT-2 inh. as adjunctive to insulin therapy in T1DM were performed [137,138,139,140,141,142]. At the end of these studies HbA1c decreased only by −0.25 and 0.52% and weight reduction ranged between −2.2 and −4.4 kg. Notably, treatment with SGLT-2 inh. was associated with increased risk of diabetic ketoacidosis [139]. The risk of this serious complication is also higher in T2DM patients treated with these agents than in those receiving placebo [143]. Moreover, genital mycotic infection and volume depletion-related events may also develop during therapy with SGLT-2 inh. Another limitation of the usage of this group of the newer hypoglycemic medications is their suggested association with bone fracture and lower limb amputation. However, the data on these complications is inconclusive and appears to be related to a specific representative of this class of drugs (canagliflozin) [144].

### 4.4. Gestational Diabetes (GDM)

Another advantage of metformin over newer hypoglycemic agents is its increasing use in the treatment of glucose metabolism disturbances in pregnancy [145]. This is because the results of numerous clinical studies indicate that metformin is an effective, safe and cheap option for women with gestational diabetes and T2DM in pregnancy and may improve maternal and perinatal outcomes [145,146,147]. The positive results of The Metformin in Gestational Diabetes [MiG] and MiG TOFU, showing that metformin had similar pregnancy outcomes to insulin therapy with less maternal weight gain and a high degree of patient acceptability, have a great impact on the current medical practice in many countries [148,149]. A meta-analysis of eight clinical trials involving 1712 pregnant women with GDM have proven that metformin and insulin therapy have similar impact on glycemic control. Interestingly, metformin treatment was associated with a lower incidence of neonatal hypoglycemia and neonatal intensive care admission [150].

Metformin is classified as pregnancy category B medication and if there is a clinical need for it, it is considered safe. Unlike metformin, all newer hypoglycemic medications are classified as pregnancy category C medications and are not used in the treatment of GDM and T2DM in pregnancy because of concern about serious adverse effects.

### 4.5. Polycystic Ovary Syndrome (PCOS)

Metformin is the only anti-diabetic drug that is used in women with PCOS for the treatment of metabolic derangements. The syndrome is often associated with obesity, IR and hyperinsulinemia, and other reproductive and metabolic disturbances [151,152]. Metformin, by increasing insulin sensitivity in target organs, can correct to some extent these abnormalities and reduce the risk of glucose intolerance as well as reducing the level of androgens, and controlling the menstrual cycle of women with PCOS [151,152,153,154]. It has been reported that metformin, besides its antihyperglycemic effect, has a positive influence on bleeding disorders in women with PCOS and has significant ovulation stimulatory effect compared with placebo [155].

A meta-analysis of 13 studies involving 1606 pregnant women with PCOS performed by Zeng et al. has shown that metformin treatment can improve clinical pregnancy rate and decrease the possibility of preterm delivery [154]. It may also reduce the risk of pregnancy-induced hypertension, early pregnancy loss, increase vaginal delivery and live birth rate. Moreover, metformin use in pregnant women decreases elevated blood glucose levels without increasing the risk of hypoglycemia [154]. The most recent meta-analysis published in 2020 has shown that use of metformin is associated with less frequent GDM development than control diets [156]. Although metformin is not approved for PCOS management, it is still the most commonly prescribed medication alone or in combination with clomiphene in women with PCOS [155,157].

In the literature available only one small, short-term study assessing the effects of empagliflozin in obese women with polycystic ovary syndrome was found [158]. The results obtained showed that treatment with this agent significantly improved the anthropometric parameters and body composition without changes in hormonal or metabolic parameters. The results of a short-term study involving 36 obese women with PCOS suggest that liraglutide may reduce body weight and abdominal circumference and eating behavior [159]. Recently, a systematic review and meta—analysis have shown that GLP-1RAs were more effective in improving insulin sensitivity and reducing body weight and abdominal girth than metformin. However, the authors of this publication underline several limitations of the studies included in the meta-analysis [160].

It has been reported that dipeptydyl peptidase-4 that has an impact on adenosine deaminase activity, Anti Mullerian Hormone and insulin levels as well as on IR [161]. Interestingly, Ferjan et al. showed in a pilot randomized study that sitagliptin may prevent weight regain in metformin intolerant obese women with PCOS [162]. The same authors reported that sitagliptin in combination with metformin prevented weight regain more effectively than metformin [163]. Therefore, further studies are warranted to prove a possible therapeutic benefit and advantage of these newer groups of hypoglycemic agents over metformin in the management of PCOS.

### 4.6. Non-Alcoholic Fatty Liver Disease (NAFLD)

A high proportion of people with T2DM also have NAFLD, as both diseases are closely associated with obesity and both are independent classical risk factors for cardiovascular major events. In preclinical studies metformin has been shown to improve NASH and decreases hepatocyte lipid synthesis with subsequent reduction of triglyceride accumulation [164]. Clinical studies, though not all of them, have reported that when metformin is used for the treatment of T2DM in people with obesity, it significantly reduces body weight, limb, android and gynoid fat mass while increasing the total lean mass. Moreover, metformin may correct several components of metabolic syndrome such as impaired glucose tolerance, lipid metabolism disturbance and reduces alanine transferase serum levels. Although, these studies suggest that metformin might be beneficial in the treatment of NAFLD, exercise and caloric restriction are the only approved treatment options acceptable for NAFLD. Nevertheless, metformin is frequently prescribed off-label to patients with this disease, because it is believed that activation of AMPK is associated with a plethora of beneficial effects, including decreasing oxidative stress and inflammation of the liver [165]. Only a few small -scale clinical trials with SGLT2 inh and GLP-1Ras in individuals with NAFLD have been conducted with some promising results [166,167]. Therefore, further studies are needed to assess their real role in this frequent health problem of the modern society.

### 4.7. Cardiovascular Protection

Diabetes exacerbates the dynamics of atherosclerosis and it is estimated that about two-thirds of deaths in people with diabetes are due to cardiovascular disease, of which approximately 40% are from coronary artery diseases [CAD], 15% from other forms of heart disease, principally heart failure [HF], and about 10% from stroke [168,169,170,171,172]. Therefore, minimizing the risk of these life-threating complications should be a prioritized strategy of treatment and clinicians should be convinced that the hypoglycemic agent which they are going to prescribe to the patient has proven cardioprotective proprieties. In 2020 we have evidence from CVOTs for SGLT2-inhibitors and GLP1-agonists that they are both effective glucose-lowering and cardio-renoprotective drugs (ADA/EASD guidelines 2020).

The cardioprotective effects of metformin were demonstrated in a series of preclinical studies. The results of 27 animal studies of experimental myocardial infarction were subjected to meta-analysis by Hessen et al. The data obtained suggest that metformin significantly limits infarct-size, reduces postinfarction remodeling and improves cardiac function in animals. However, the authors of this meta-analysis emphasize the methodological shortcomings, risk of publication bias and substantial between –study heterogeneity. All this makes it difficult to formulate an unambiguous opinion about the cardioprotective effect of metformin [173].

The current knowledge indicates that the mechanism responsible for the cardioprotective action of metformin is complex and multidirectional [173,174,175]. Molecular research shows that metformin exerts both AMPK–dependent and AMPK-independent effects on the cardiovascular system [175,176]. Restoring the glucose uptake by cardiomyocytes and switching their metabolism from lipid utilization to glucose utilization, metformin significantly contributes to the improvement of cell mitochondrial respiration and ATP synthesis in failing heart and determines the effectiveness of the systolic and diastolic function of the heart [177,178,179]. AMPK activated by metformin in endothelium cells promotes their integrity, stimulates the production of nitric oxide and reduces oxidative stress [178,180]. Moreover, the agent has the ability to reduce the level of vascular cell adhesion molecules (ICAM-1 and VCAM-1), inhibits conversion of monocyte to macrophage with decreased secretion of pro-inflammatory cytokines and reduced monocyte adhesion to endothelial cells with decreased secretion of proinflammatory cytokines [181,182]. 

Metformin may protect against the negative effects of angiotensin II on cardiovascular system, inhibit the synthesis of the plasminogen activator inhibitor type 1 (PAI-1), reduce platelet aggregation as well as transforming growth factor-β and preventing the opening of the mitochondrial permeability transition pore and, finally, regulating calcium turnover in cardiomyocytes [178,183,184,185,186]. It has also been reported the metformin elevates the expression of endothelial nitric oxide synthase (eNOS) and peroxisome proliferator –activated receptor –gamma coactivator-1 alfa in cardiomyocytes [179]. The agent may also reduce myocardial cell apoptosis and promote autophagy. Activation of AMPK and improving signaling pathway of this enzyme by metformin increases insulin sensitivity of cardiomyocytes with all positive results of this effect, including improvement of glucose and lipid metabolism in in these cells [174,176,178,187].

It should also be pointed out that metformin inhibits adipocytes differentiation and hypertrophy and reduces adipose tissue inflammation, hence potentially decreases the risk of cardiac fibrosis and diminishes the density of micro-arteries leading to structural changes and systolic and diastolic dysfunction of the myocardium. Moreover, the agent may positively change leptin to adiponectin rate in pericoronary fat in patients with acute myocardial infarction [174,178,187,188,189,190].

The mechanisms of cardiovascular effects of metformin, described briefly above, are supported by clinical studies assessing the influence of the drug on the development and progression of atherosclerosis-related diseases, including CAD, in people both with and without T2DM. Interestingly, metformin seems to be cardioprotective both in people with short and long duration of the disease [4,11,191]. Notably, metformin treatment results in the inhibition of atherosclerosis progression also in people with type 1 diabetes [192].

**Figure 2:**
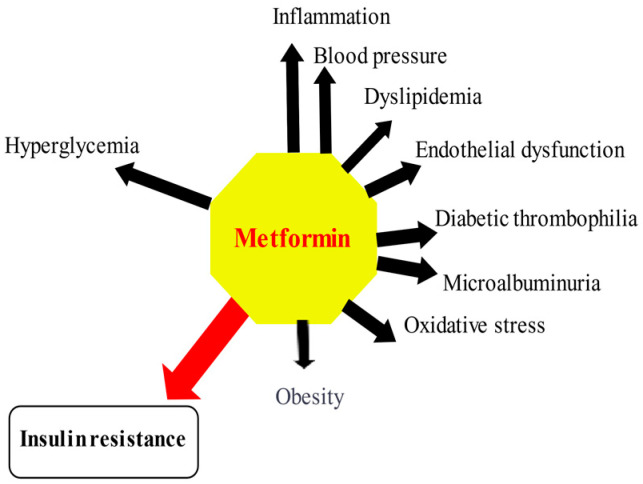
Metformin has multifactorial beneficial effect on cardiovascular risk factors. Note that metformin reducing IR leads to the reduction of other cardiometabolic risk factors.

Most, although not all, clinical studies have shown that metformin, lowering IR, may reduce the risk of chronic vascular complications of diabetes and significantly decrease modifiable cardiovascular risk factors, including hyperglycemia and hyperglycemia -related inflammation and oxidative stress, increased platelet aggregation PAI, HbA1c, dyslipidemia, IR, visceral obesity, and blood pressure [192,193,194]. The effect of metformin on cardiovascular risk factors is depicted in Figure 2.

The evidence from multiple studies and from everyday practice indicates that the level of HbA1c– the most reliable marker of glycemic control- positively correlates with the risk of cardiovascular complications and is an independent risk factor for CVD, especially CAD and ischemic stroke [195,196,197,198]. In the UKPDS study HbA_1c_ levels were the third most important factor, behind dyslipidemia and hypertension, in determining cardiovascular risk in T2DM patients [168].

Stratton et al. evaluated the relation between exposure to glycemia over time and the development of macrovascular and microvascular complications finding that 1% reduction in updated mean HbA_1c_ was associated with reductions in risk of 21% for any end point related to diabetes, 21% for deaths related to diabetes, 14% for myocardial infarction, and 37% for microvascular complications. Based on these results it was concluded that any reduction in HbA_1c_ is likely to reduce the risk of diabetes complications, with the lowest risk being in people with HbA_1c_ values of <6.0% [199]. Therefore, an elevated level of HbA1c should be reduced to the target without risk of hypoglycemia and agreed between the doctor and patient as soon as possible. The ability of the newer, particularly oral hypoglycemic agents (SGLT-2 inh. and DPP-4 inh) to reduce the value of this biomarker is weaker than that of metformin which decreases HbA1c by approximately −1.2 and 1.5% [200,201,202].

The stronger antihyperglycemic effect of metformin without increasing the risk of hypoglycemia and shortening the time of exposure to high blood glucose level seems to favor this agent over newer ones in the initiation of pharmacotherapy of T2DM, at least in patients without established cardiovascular disease or cardiovascular risk factors (primary prevention). However, metformin is also effective in the secondary prevention of atherosclerosis associated diseases in T2DM patients [199,203,204,205,206].

The preventive effect of metformin under real world conditions in monotherapy or in combination on the cardiovascular system has been shown both in clinical trials and in primary care settings. Here are some examples: Eurich et al. demonstrated in a large group of subjects treated with metformin alone or in combination, that the mortality rates among T2DM and HF patients were substantially lower than in sulfonylurea users. Moreover, they did not observe increased hospitalization and death rates attributable to metformin-induced lactic acidosis [207]. Similarly, Aguilar et al. observed in a study on 6185 ambulatory patients with HF and diabetes that metformin users had significantly lower rates of mortality than non-users and total hospitalization rate was not significantly different between the two compared groups. 

Mohan et al. conducted a randomized controlled trial involving 68 patients with diagnosed coronary artery disease with IR and/or prediabetes assessing the effect of a 12-month treatment with metformin XL in doses of 2000 mg daily or placebo on the left ventricular hypertrophy. They found that metformin significantly reduced left ventricular mass and left ventricular mass indexed to height. Moreover, the drug decreased systolic blood pressure, body weight, and oxidative stress [208]. Interestingly, Lexis et al. showed that prolonged metformin treatment of T2DM patients in the case of MI with ST-segment elevation MI reduced its size in comparison with non-metformin users [209]. The REACH (Reduction of Atherothrombosis for Continued Health) study showed that treatment with metformin of patients with atherothrombosis was associated with a 24% reduction of mortality compared with no metformin regime [3]. These observations are in agreement with the results of the very first systematic review of eight studies conducted by Eurich et al. [210]. The aim of this review was to determine whether there is any association between hypoglycemic agents and morbidity and mortality in diabetics with HF. Based on the data obtained, the conclusion was drawn that—when compared to other anti-hyperglycemic therapies—metformin significantly reduced mortality and hospital admissions. These effects were noted despite the similar decrease in HbA_1C_ values, confirming previous suggestion that metformin may have additional beneficial effects beyond the blood glucose lowering property [210]. Six years later Eulrich et al. confirmed previously obtained data in a new systematic review of observational studies involving 34,000 patients with T2DM and HF [211]. In another meta-analysis of 53 studies published in 2017 by Campbell et al. it was proved again that metformin significantly reduces the risk of all-cause mortality. The authors suggested that the agent may decrease the risk of ageing-related diseases (i.e., cardiovascular disease, cancer] but the effect was independent of its hypoglycemic activity [211].

Very recently, Han et al. published a systematic review and an updated meta-analysis assessing the relationship between metformin and cardiovascular complications. To accomplish this task they examined 40 clinical trials comprising 1,066,408 patients with diagnosed CAD. The findings of this meta-analysis strongly support the experience over decades of many clinicians and researches that metformin may significantly decrease cardiovascular mortality, all-cause mortality and CV events, defined as recurrent MI, HF, recurrent angina, atrial fibrillation (AF), malignant arrhythmia, cardiac death in patients with MI and HF. Furthermore, it was found that besides the anti-hyperglycemic effect, metformin could reduce all-cause mortality in the T2DM subgroup of patients with HF only [6]. 

Considering the cardioprotective potency and safety of metformin, a recently published of 17 observational studies is of particular interest. The aim of this work was to compare the effect of diabetes regimens that included metformin to regimens without metformin on prespecified clinical outcomes in people with T2DM coexisting with HF and moderate to severe chronic kidney disease (CKD) or chronic liver disease (CLD). It was found that metformin use in patients with T2DM and historical metformin contraindications (CKD, HF or CLD) was associated with lower all-cause mortality. Furthermore, a lower rate of hypoglycemia was registered in the metformin group than in the compared group [212]. These findings are in accordance with the results of other studies [211,213,214].

However, in contrast to the studies suggesting a beneficial impact of metformin on the structure and function of the left ventricle, the findings from sub-analysis of the Glycometabolic Intervention as adjunct to Primary Coronary Intervention in ST-Elevation Myocardial Infarction [GIPS-III Study] did not confirm metformin’s ability to improve diastolic function of the left ventricle [215]. The uncertainty about the substantial cardioprotective effect of metformin made Griffin et al. perform a meta-analysis of randomized trials and found that metformin treatment reduces, albeit insignificantly, the risk of all-cause mortality in diabetics (−4%), cardiovascular death (−3%), MI (−11%), peripheral vascular disease (−19%) and increases the risk of stroke (+4%). The emerging doubts will be difficult to dispel because conducting a large, long-term study assessing the effect of metformin monotherapy on the cardiovascular system is, for many reasons, extremely difficult to implement [9].

However, despite the opinions questioning the cardioprotective effects of metformin, a large number of diabetologist and general practitioners, based upon their positive experience, prescribe this particular agent to their patients with newly-diagnosed T2DM. When considering the possibility of replacing metformin with the newer antidiabetic agents as the first-line treatment of T2DM, it is worth noting that there is a lack of data on the long-term cardiovascular effect of these medications. The average duration of CVOS was around five years while the effect of metformin on chronic vascular T2DM complications had been observed in the UKPDS Effect of [1,2] over two decades and in some clinical trials 2–3 times longer. The studies with newer generation of hypoglycemic medications were performed in patients with a long history of the disease and already had cardiovascular disease or a number of risk factors for it. However, it should be underlined that the substantial proportion of people with newly-diagnosed T2DM, particularly young, are often without cardiovascular disease or risk factors, except hyperglycemia. That is why we agree with Griffin et al. who assume that “metformin will hold its leading position in the management of hyperglycemia in diabetes for difficult to foresee period of time” [9].

### 4.8. Nephroprotection

The prevalence of CKD has increased worldwide mainly as a result of the rapidly increasing number of people with diabetes [216,217]. CKD attributed to diabetes (diabetic kidney disease, DKD) affects approximately 30% of people with T1DM and about 40% with T2DM. This microvascular complication of chronic hyperglycemia often leads to end-stage renal disease and significantly increases the risk of cardiovascular disease and premature death. Therefore, renoprotection as well as cardioprotection remain the major therapeutic challenge in diabetes. Unfortunately, the current methods of pharmacological treatment of DKD are not effective enough and many patients will need, sooner or later, dialysis or kidney transplantation. 

Recently, several CVOTs with the newer hypoglycemic medications have shown that, beyond the anti-hyperglycemic effect, these agents significantly reduced cardiovascular risk and prevented the development and progression of albuminuria and lowered the decline in estimated glomerular filtration rate [eGFR) [218,219,220,221,222,223]. DPP-4 inhibitors are considered to have neutral or in some studies beneficial renoprotective effects [220,224]. Therefore, the ADA and EASD recommend early initiation of combination therapy with some never agents in T2DM assuming that it may significantly increase the probability of achieving the glycemic target for a specific patient and significantly reduce the risk of chronic cardiorenal complications [225]. In spite of the very promising results of clinical trials indicating renoprotective effects of some newer antidiabetic drug, uncertainty remains as to whether they are superior or equivalent to metformin. To answer this question is not easy due to the lack of direct long-term observational comparative studies with the newer hypoglycemic drugs and metformin. Evidence from a series of experimental studies suggests that metformin has nephroprotective properties attenuating kidney injury produced by different toxins, including exposure to high glucose concentration [226,227].

The molecular mechanism of renoprotective effects of metformin is complex and not fully clarified. However, multiple data indicate that the key role is played by the activation of AMPK and subsequent improvement of mitochondrial biogenesis and a whole cascade of beneficial effects induced by this enzyme in kidney cells [228,229]. This hypothesis is supported by the observations indicating that the activity of AMPK in the kidney of diabetic rats was reduced and metformin could repair this abnormality trough the inhibition of oxidative injury [229].

The impact of metformin on the kidney in diabetics is also a consequence of both its glucose lowering-dependent and glucose-independent mechanism [230]. The toxic effect of hyperglycemia on the kidney is well recognized both in animal models and in humans. Hyperglycemia increasing oxidative stress and generation of free radicals, inducing low-grade chronic inflammation, and autophagy is responsible for podocyte damage and its death and for the intensification of tubule-interstitial fibrosis. Therefore, metformin with its potent anti-hyperglycemic effect, inhibition of advanced glycation end products accumulation in various tissues and decreasing hyperglycemia -related inflammation and oxidative stress may slow down all these kidney damaging processes [229,230,231]. Experimental studies on mammalian cells have shown that metformin increases the level of anti-aging protein (klotho protein) which may inhibit the progression of various kidney diseases. In addition, the drug has an ability to decrease the level of the mammalian target of rapamycin [mTOR] protein and reverse the effect of hyperglycemia on the activity of Madin -Derby Canine Kidney cells (MDCK cells) [232]. Another study demonstrated that in rat diabetic nephropathy model metformin increases the expression of anti-oxidative genes and inhibits pro-inflammatory genes [233]. It was also shown that metformin, in addition to the inhibitory effect on oxidative stress and inflammation, inhibits ER stress and related mesangial expression of transforming growth factor and production of extracellular matrix and induces autophagy [230,233]. However, the renoprotective effects of metformin has been questioned by some researchers. Lee et al. observed a selected group of T2DM patients enrolled under the pay -for—performance program of the National Health Insurance in Taiwan. The aim of that study was to assess the impact of metformin on the development of end-stage kidney disease and CKD. The findings indicate that among metformin users vs. non-users the risk of the end-stage kidney disease and CKD development was 22% and 25% higher, respectively [234].

Numerous clinical studies have demonstrated that metformin decreases insulin resistance and improves intracellular signaling of insulin, reduces glucotoxicity and lipotoxicity. Hyperglycemia and diabetic dyslipidemia are among the major risk factors of CKD development and progression. Therefore, it is rational to expect that metformin—by reducing the mean HbA1c by approximately 1.0–1.3% and improving lipid profile and when implemented soon after T2DM diagnosis—will attenuate the risk of chronic diabetes complications, including kidney impairment [74]. The post-interventional phase of the UKPDS showed a 16% reduction in microvascular complications of T2DM, defined as vitreous hemorrhaging, retinal photocoagulation, or renal failure [2]. These results are in line with the data presented in a recent retrospective study conducted on a very large group of T2DM patients with DKD stage 3. It has been shown that long-term metformin use was associated with 35% and 33% risk reduction in all-cause-mortality and end-stage renal disease progression, respectively. It is especially important that metformin usage did not increase the risk of lactic-acidosis [235]. Interestingly, Stephen et al. observed that many kidney transplant recipients were safely treated with metformin. No association between metformin use and worsening of patient’s condition or allograft survival was found [236].

Recently, evaluation of data from a cohort study of the Swedish National Diabetes Register with 51,675 patients aged from 40 to 85 years with T2DM and different levels of kidney function receiving metformin therapy or any other antidiabetic treatment in outpatients’ clinics and primary healthcare clinics showed no increased risk of CVD and all-cause mortality. Lactic acidosis and serious infection did not develop in patients with eGFR 30–45 mL/min/1.73 m. The authors of this study concluded that the benefits of metformin use clearly outbalance the risk of severe side effects [237].

Charytan et al. showed that metformin treatment in people suffering from T2DM and CKD was associated with a reduced risk of all-cause mortality and cardiovascular death by 51%. Moreover, the drug decreased the cardiovascular composite endpoint and the kidney disease composite endpoint by 23% [238].

Bell et al. performed a retrospective analysis of the electronic health records of 25,148 T2DM patients, of whom 14,622 were treated with metformin at some point during the study period and 4944 of the entire cohort had at least one episode of acute kidney failure between 2004 and 2013. Interestingly, they found that the use of the drug did not increase the risk of acute kidney disease. Moreover, the drug reduced all-cause mortality and prolonged survival in patients with diabetes and CKD treated previously with this agent [239]. These observations are in line with the results of a large observational study including 469,688 T2DM patients treated in general practice. It was found that metformin decreased the risk of severe kidney failure compared with sulfonylureas and insulin use [240]. Metformin’s renoprotective effects have also been strongly supported by the aforementioned systematic review by Crowley et al. They examined 17 observational studies comparing the effect of diabetes regimens that included metformin to regimens without metformin on prespecified clinical outcomes in people with T2DM coexisting with HF, CKD or chronic liver disease. The results obtained showed that metformin exposure in patients with T2DM and the presence of metformin contraindications (moderate CKD, HF or chronic liver disease) was associated with lower all-cause mortality and fewer HR readmissions in diabetics with CKD or HF. Furthermore, a lower rate of hypoglycemia was found in the metformin group than in the comparison group [212]. 

A case control study performed in Lodz, Poland showed also that in spite of classical contraindication 275 out of 558 hospitalized diabetics had been treated for a long time before admission, with metformin alone or in combination with other hypoglycemic agents. Despite contraindications none of them reported any serious adverse effects and no significant pH changes were observed. Only three patients reported moderate dyspepsia [241].

For a long time metformin has been contraindicated in moderate renal impairment because of fear of the increased risk of serious metformin associated lactic acidosis (MALA). However, long term experience with metformin use and data available from real-life have proven that the drug is safe in such clinical situation. Observation of 50,048 T2DM patients who were treated with different oral hypoglycemic agents revealed that a very small number of them had developed MALA. The estimated incidence was only 3.3 cases per 100,000 person-years among metformin users [242]. These observations were confirmed, among others, by pooled data from 347 cohort studies which indicated that the incidence of MALA ranged from 4.3 to 5.4 per 100,000 patient-years in the group of patients treated with metformin. The authors conclude that there is no evidence from prospective comparative trials or from observational cohort studies that metformin is associated with an increased risk of lactic acidosis, or with increased levels of lactate, compared to other anti-hyperglycemic treatments [243]. 

According to the FDA Drug Safety Communication published in April 2016 metformin can now be initiated in patients with stable eGFR /1.73 m^2^; however, it should not be started in those with an estimated glomerular filtration rate (eGFR) of 45 to 60 mL/min/1.73 m^2^. Reduction in total daily dose and close kidney function monitoring are recommended in patients with eGFR between 30 and 45 mL/min/1.73 m^2^, and due to the risk of MALA, it should not be used in patients with eGFR < 30 mL/min/1.73 m^2^. It is also worth underlining that due to the potential risk of the acute kidney impairment after intraarterial iodinated contrast administration, metformin should be stopped prior this procedure only in patients with an eGFR below 60 mL/min/1.73 m^2^ or in patients with a history of liver disease, alcoholism, or heart failure. eGFR should be checked after 48 h, with metformin restarted if kidney function is stable. 

### 4.9. Cancer

People with diabetes, particularly with T2DM, have increased risk of developing specific types of cancer (liver, pancreas, endometrium, colon and rectum, breast, bladder) and death from cancer [36,97,244,245,246,247]. The possible relationship between these life-threatening diseases comprises hyperglycemia, hyperinsulinemia, obesity and IR and several others non-modifiable risk factors (age, sex, race/ethnicity) and modifiable risk factors (obesity, diet, physical activity, tobacco smoking and alcohol consumption [244].

Metformin exhibits clinically desirable effects on glucose and insulin concentration in the blood, IR and body weight. Therefore, it is not surprising that there is an interest in using this cheap, available and well tolerated agent in cancer prevention and therapy, especially that there is a continuously increasing number of clinical studies suggesting the effectiveness of metformin in both glucose control and cancer prevention [248]. 

It is interesting that since the first publication suggesting that metformin may decrease the risk of cancer development in T2DM patients, the underlying mechanism of its antineoplastic suppressing activity has remained only partially understood [97,245,247,249,250]. The current knowledge proposes at least two routes of metformin action which may contribute to its anti-cancer properties. An indirect action on tumor cells is related to metformin glucose lowering effect, reduction of hyperinsulinemia, insulin-like growth factor-1 (IGF-1) and IR [160,161]. These changes may inhibit cancer cells proliferation through limiting supplementation of energy (glucose) for cancer cells. Through this route metformin may also reduce the level of proinflammatory cytokines, and an increase of immune response to cancer cells, both of each may play a role in tumorigenesis [152,244,248]. 

Several molecular mechanisms responsible for direct anticancer activity of metformin have been suggested. They include activation of AMPK in tumor cells or suppression of: mammalian target of rapamycin (mTOR), mitogen-activated protein kinase (MAPK), protein kinase B (Akt), insulin//IGF-1 axis, NFκB pathways, and inhibition of complex 1 of the mitochondrial electron transport chain [36,97,98,245,246,247,248,249,250,251,252,253,254,255,256] Furthermore, it was reported that metformin suppresses epithelial to mesenchymal transition and directly kill stem cells [257]. Mechanism of metformin action in cancer is extensively reviewed in ref. [251,252,253,255].

These briefly presented molecular effects of metformin on cancer cells have created the hope that this agent may be useful in the prevention and treatment of specific types of tumors in humans. This suggestion has been reinforced by the results of retrospective observational studies as well as by reviews and meta-analyses [246,258,259]. Here are some examples. Nearly two decades ago Evans et al. conducted a controlled case control study of cancer incidence in patients with newly diagnosed T2DM who had or had not received metformin. The risk of cancer in the metformin group was reduced compared to non-metformin taking group. Interestingly, a trend towards a greater protective effect was observed with increasing duration of exposure to metformin [246]. A significant risk reduction of pancreatic and hepatocellular cancer incidence in diabetic patients receiving metformin was noted in a meta-analysis performed by Decensi et al. [259]. However, the more recent studies assessing the role of metformin in cancer prevention and treatment have produced mixed results. In the first observational prospective study with a median follow –up of up to 9.6 years. Gijs et al. found that in patients taking metformin compared with patients not taking metformin, the adjusted hazard ratio for cancer mortality was 0.46 [260]. Coyle et al. performed a meta-analysis of 27 eligible observational studies assessing the adjuvant effects of metformin in patients with early—stage colorectal cancer or with early stage prostate cancer on chemotherapy or radiotherapy. The findings indicate that metformin use in patients with early stage colorectal or prostate cancer compared with non-use was associated with a significant benefit in all outcomes (recurrence free survival, overall survival and cancer-specific survival). Interestingly, no benefit was observed in patients with breast and urothelial cancer [111]. These observations suggest that metformin can be an useful adjuvant agent after chemotherapy or radiotherapy of specific types of cancer. Numerous experimental and clinical studies have revealed that metformin increases sensitivity to chemo- and radiotherapy [261,262,263]. Hirsch et al. provided evidence that metformin has the ability to selectively kill cancer stem cell. Thus, it is likely to increase the effect of chemotherapeutic drugs on tumor mass and prevents relapse [264].

Jiralerspong et al. assessed the effect of metformin on pathologic remission response (pCR) in diabetic patients with breast cancer receiving three to six courses neoadjuvant anthracycline based chemotherapy. The obtained results showed that those patients who were treated with metformin during neoadjuvant chemotherapy had a higher pCR rate than metformin non-users [263]. The findings from the meta-analysis of 17 retrospective cohort studies comprising 14,333 patients with various cancer types and diabetes showed that metformin appears to improve tumor response to radiotherapy. Furthermore, 2-year and 5-year overall survival rate was higher in metformin users than in non-users. However, the authors of this publication emphasize that these results must be interpreted with caution because of the fact that not all the studies were randomized [265].

A recently published systematic review and meta- analysis have confirmed that monotherapy of T2DM with metformin was associated with a significantly lower risk of cancer incidence than monotherapy with sulfonylurea [266]. A survival benefit associated with metformin treatment in people with cancer and T2DM compared with treatment with other hypoglycemic agents was found in the meta-analysis done by Yin et al. Therefore, the authors conclude that metformin should be the drug of the first-line in the management of such patients [267]. On the other hand, evaluating the electronic records of 315,890 people aged 21–87 years with incident diabetes Danker et al. did not find an inverse association between metformin use and the risk of major cancer event [268]. The inconclusive results regarding the role of metformin in cancer prevention and treatment in people with diabetes justify further on-going randomized studies.

### 4.10. Longevity

Another argument supporting the leading position metformin among currently available antidiabetic drugs is its impact on the life-span. It is rational to assume that any glucose-lowering drug decreasing the risk of cardiovascular disease, renal failure or cancer will have a positive effect on the life-span. However, with the spectacular results of CVOTs, it can be expected that the newer hypoglycemic drugs will extend the lives of people treated with these medications. Unfortunately, there are not enough long-term observations that could confirm this, while in the case of metformin, there is evidence from some, but not all, preclinical experiments indicating that this agent can extend longevity in worms and mice [269,270]. These observations have been at least partially confirmed in some retrospective clinical observations. They have shown that administration of metformin is associated with a reduced incidence of several age-related disease. Bannister and al compared all-cause mortality in a large UK register of T2DM patients treated with metformin monotherapy or sulphonylurea respectively. with matched individuals without diabetes. They found that people initiated with metformin in monotherapy had longer survival than those receiving sulphonylourea as a first line treatment. Interestingly enough, the study also showed that patients with T2DM taking metformin had longer survival than people without diabetes not taking this drug [271].

Recently, Campbell et al. presented a systematic review of 53 publications assessing the effect of metformin on all-cause mortality and diseases of aging. The findings showed that metformin using diabetics had significantly lower all—cause mortality then non users and non—diabetics. People with T2DM treated with metformin also had lower all-cause—mortality than those receiving sulphonylurea or insulin. Additionally, metformin reduced cardiovascular disease in diabetics taking metformin compared to non-takers and decreased the risk of malignancies compared to non-diabetics. Based on these results the conclusion was made that metformin could extend the life and health span by acting as an anti-aging therapeutics in humans [272].

### 4.11. COVID-19

Patients with diabetes are at higher risk from COVID-19 disease by increased inflammatory activity and vice versa COVID-19 increases the risk of diabetes and accelerate its progression [273,274,275,276]. Interestingly enough another unique property of metformin was signaled by the results of a retrospective study evaluating the effect of this drug on the risk of mortality in 6256 patients (3302 F) aged ≥ 18 with T2DM or obesity and hospitalized between 1 January and 7 June 2020 for COVID-19 confirmed by polymerase chain reaction [277]. The patients included in the study were assigned to metformin home users’ group (at least 90 days of metformin prescription from 12 months prior COVID-19 diagnosis) or non-users before hospitalization. It was found that of the 2333 people in the metformin group, 18.9% died vs 21.3% of 3923 who died in the non-users group. These results suggest that metformin use before hospitalization (metformin use is discontinued at hospitalization in the USA) was not associated with significantly decreased mortality in the overall sample of the patients. However, significantly reduced mortality was noted in women with T2DM or obesity who were admitted to hospital for COVID-19 (OR 0.79, 95% CI 0.64–0.98, *p* = 0.03) [277].

As mentioned earlier, it has been reported that both obesity and diabetes are associated with chronic inflammation and that metformin reduces the level of pro-inflammatory biomarkers and increases secretion of anti-inflammatory cytokines [35,38,39,105,106]. Therefore, the authors of this study suggest preventive use of this agent before infection with SARS-CoV-2 by people with diabetes and/or obesity while emphasizing an urgent need for further research on this intriguing and particularly important topic. Considering the devastating impact of COVID-19 pandemic on the world’s population the results of the study presented above strengthen the position of metformin among antidiabetic drugs.

## 5. Pharmacogenetics of Metformin

Long-term clinical experience supported by numerous clinical studies clearly indicates that a substantial percentage of T2DM patients do not achieve the expected level of glycemic control while receiving metformin in monotherapy [278]. Considerable intra-individual variability in metformin response may be caused by numerous factors such as: age, gender, physical inactivity, insufficient dose of the drug, non-adherence, interaction between metformin and other drugs and metformin interaction with microbiota [279,280]. Experimental and clinical studies in both healthy volunteers and diabetic patients have shown that specific groups of genes (e.g., the family of SLC genes) are involved in metformin absorption, distribution, metabolism and elimination and, as a result, may affect the pharmacological response to this drug [281,282,283,284,285]. It has also been demonstrated that the polymorphisms of many genes may increase or decrease the therapeutic response to metformin [286] The most thoroughly investigated gene was SLC22A1 encoding an organic cationic transporter 1(OCT1) which is localized in hepatocytes. OCT1 is responsible primarily for the uptake of metformin by the liver—a main target of the drug action on glucose metabolism. However, the role of SLC22A1 polymorphisms in response to metformin has not been fully clarified [283,286,287]. As has recently been demonstrated in a systematic review, which assessed the association between OCT1 polymorphisms and the biochemical and clinical outcomes in metformin users, only some of this gene’s polymorphisms were associated with the variable response to the drug [288]. Apart from OCT1, various other transporters, play a role in metformin kinetics, including the multidrug and toxin extrusion 1 (MATE1), encoded by the gene SLC47A1 and MATE2-K, encoded by the gene SLC47A2. These transporters regulate the renal clearance of metformin [281,289]. The impact of these transporters on metformin elimination may contribute to a marked elevation of the drug concentration, particularly in the liver, increasing the risk of lactic acidosis [290].

The absorption of metformin from the gut is primarily mediated by plasma membrane monoamine transporter (PMAT), encoded by the gene SLC29A4 [291]. However, there is lack of clinical data on the impact of PMAT on pharmacokinetic parameters of metformin in human.

Dujic et al. performed a large-scale meta -analysis across 10 international cohorts of the Metformin Genetics Consorciun (MetGen) to clarify the significance of nine candidate polymorphisms in five transporter gene (OCT1–3), MATE 1 and OCTN1 in glycemic response to metformin. The findings from almost 8000 participants suggest that variations in these transport genes contribute little to variability on the glycemic response to metformin in T2DM. This important study provides evidence that despite the well-recognized role of cation-selective transporters in metformin pharmacokinetics, changes in these transporters have no significant impact on glycemic response to metformin. Therefore, there is uncertainty whether genotyping of metformin transporters could be used in personalized metformin therapy. This can be elucidated in further studies involving large populations [292].

## 6. Safety of Metformin

Extensive worldwide experience with the use of metformin in an uncountable number of T2DM patients supported by numerous clinical studies clearly indicates that metformin is safe and generally well-tolerated. However, in 20–30% of patients the drug may induce gastrointestinal disturbance, mostly mild or moderate, such as dyspepsia, dysgeusia, heartburn, abdominal pain, nausea, vomiting, bloating or diarrhea [293]. These symptoms may have an unwanted impact on the quality of life and treatment adherence. However, in the majority of patients, they often disappear with time or with the use of extended—release pills, gradual dose titration, and metformin administration with meals. Nevertheless, in approximately 5% of patients severe symptoms of gastrointestinal intolerance develop. They are most likely related to high concentration of metformin in the intestine, increased secretion of serotonin from enterochromaffin cells, decreased ileum absorption of the bile acids, increased secretion of GLP-1 from enterocytes and altered microbiome. All these factors have a substantial influence on gastrointestinal regulation, particularly intestinal motility and secretion [294]. 

It has been reported that in 6–30% users, metformin is responsible for vitamin B12 malabsorption and/or deficiency. It is well-known that the low level of vitamin B12 increases the risk of megaloblastic anemia and peripheral neuropathy. These complications develop more frequently in the elderly patients and correlate positively with both the duration and daily dose of metformin [295]. In patients who develop these complications, B12 should be monitored and supplementation with vitamin B12 may be required [296,297,298]. 

Donnelly et al. analyzed data from A Diabetes Outcome Progression Trial (ADOPT) and UKPDS, and from real-world showed that the use of 1 g of metformin daily increased the risk of anemia by 2% per year [299]. Interestingly enough, the REMOVAL study showed that mid-age people with T1DM treated with metformin over 3 years had biochemical deficiency of vitamin B12 that was twice as high as individuals taking placebo (12% vs. 5%, respectively [192]. These findings are of clinical importance because in the younger population vitamin B12 is often associated with gastroparesis, megaloblastic anemia and coeliac disease. At least three putative mechanisms of metformin-induced deficiency of vitamin B12 have been proposed, including: (1) alteration of microbiome resulting in the binding of the intrinsic factor-vitamin B12 complex (IF-vitamin B12 complex) to bacteria and blocking its absorption into circulation, (2) altering the intestinal motility and (3) inhibition of the calcium-dependent IF-vitamin B12 complex binding to the receptor located in the terminal ileum [294]. The risk of lactic acidosis is described in the section titled “Nephroprotection”.

## 7. Conclusions

Experimental and clinical evidence indicates that metformin has a pleiotropic mechanism of action and, consequently, the drug reduces the elevated glucose level in people with prediabetes and diabetes without increasing the risk of hypoglycemia and weight gain. Moreover, metformin may reduce the development and progression of some types of cardiovascular disease and inhibit the progression of kidney damage in people with diabetes. It should also be highlighted that the historical contraindication (HR and kidney impairment) for this inexpensive, safe, effective and generally accessible drug have recently been challenged. Therefore, in most patients pharmacologic therapy with metformin should be initiated at the time of diabetes diagnosis. However, in patients with cardiovascular risk factors or cardiovascular disease combined therapy with metformin plus SGLT-2 or GLP-1RAs should be started as soon as possible. 

Metformin is also promoted as an effective medication for PCOS and used off-label in the management of T1DM, GDM and NAFLD. There are also increasing attempts to use metformin in cancer prevention and treatment as well as the reduction of mortality in people with diabetes and COVID-19 disease. This made it possible to use this remarkable drug in a much larger group of patients worldwide. However, the rationale of these observations can only be confirmed after long-term, well-designed studies have been performed. The main points of the review are presented in Table 2.

## Figures and Tables

**Table 1:** The old recommendations and new possibilities for the use of metformin.

Approved to Treat	No Formal Indication (Used Off-Label)	Investigated for New Applications
T2DM	Prediabetes/obesity	Cardioprotection
	T1DM	Nephroprotection
	GDM	Cancer
	PCOS	Anti-aging
	NAFLD	COVID-19

**Table 2:** The main points of the review.

Metformin:
Is an effective and safe antihyperglycemic agent in monotherapy as well as in combination with other anti-diabetic medicines.
Is indicated as the first -line therapy of newly diagnosed T2DM
Inhibits or delays the risk of the transition from prediabetes to T2DM
Lowers hyperglycemia mostly via inhibition of hepatic gluconeogenesis along with increasing insulin sensitivity in skeletal muscle
Suppresses hepatic glucose production through the inhibition of mitochondrial respiratory chain complex 1
Acts in AMPK—dependent and AMPK-independent manner
Shows multiple glucose-independent pleiotropic effects
Possesses cardioprotective properties
Has possible nephroprotective effect
Off-label use is increasing in PCOS, T1DM, NAFLD and obesity
Is considered to be promising agent in cancer prevention and treatment as well as an anti-aging agent
